# Identification of cell types in a mouse brain single-cell atlas using low sampling coverage

**DOI:** 10.1186/s12915-018-0580-x

**Published:** 2018-10-11

**Authors:** Aparna Bhaduri, Tomasz J Nowakowski, Alex A Pollen, Arnold R Kriegstein

**Affiliations:** 1 Department of Neurology, UCSF, San Francisco, USA; 2 The Eli and Edythe Broad Center for Regeneration Medicine and Stem Cell Research at UCSF, San Francisco, USA; 3 Department of Anatomy, UCSF, San Francisco, USA

**Keywords:** Single-cell analysis, Downsampling, Cell atlas studies, Bioinformatics

## Abstract

**Background:**

High throughput methods for profiling the transcriptomes of single cells have recently emerged as transformative approaches for large-scale population surveys of cellular diversity in heterogeneous primary tissues. However, the efficient generation of such atlases will depend on sufficient sampling of diverse cell types while remaining cost-effective to enable a comprehensive examination of organs, developmental stages, and individuals.

**Results:**

To examine the relationship between sampled cell numbers and transcriptional heterogeneity in the context of unbiased cell type classification, we explored the population structure of a publicly available 1.3 million cell dataset from E18.5 mouse brain and validated our findings in published data from adult mice. We propose a computational framework for inferring the saturation point of cluster discovery in a single-cell mRNA-seq experiment, centered around cluster preservation in downsampled datasets. In addition, we introduce a “complexity index,” which characterizes the heterogeneity of cells in a given dataset. Using Cajal-Retzius cells as an example of a limited complexity dataset, we explored whether the detected biological distinctions relate to technical clustering. Surprisingly, we found that clustering distinctions carrying biologically interpretable meaning are achieved with far fewer cells than the originally sampled, though technical saturation of rare populations such as Cajal-Retzius cells is not achieved. We additionally validated these findings with a recently published atlas of cell types across mouse organs and again find using subsampling that a much smaller number of cells recapitulates the cluster distinctions of the complete dataset.

**Conclusions:**

Together, these findings suggest that most of the biologically interpretable cell types from the 1.3 million cell database can be recapitulated by analyzing 50,000 randomly selected cells, indicating that instead of profiling few individuals at high “cellular coverage,” cell atlas studies may instead benefit from profiling more individuals, or many time points at lower cellular coverage and then further enriching for populations of interest. This strategy is ideal for scenarios where cost and time are limited, though extremely rare populations of interest (< 1%) may be identifiable only with much higher cell numbers.

**Electronic supplementary material:**

The online version of this article (10.1186/s12915-018-0580-x) contains supplementary material, which is available to authorized users.

## Background

Recent efforts seek to create a comprehensive cell atlas of the human body [1, 2]. The advent of single-cell and single-nucleus mRNA sequencing (RNAseq) in droplet format [3–5] now enables large-scale sampling of cells from any tissue, and clustering of these large-scale datasets enables cell type and subtype classification [3, 6–8]. However, the human body contains orders of magnitude more cells than can be analyzed by current technologies. Therefore, designing effective sampling strategies are critical to generate a working atlas in an efficient, cost-effective, and streamlined manner. A recently released publicly available dataset of 1.3 million single cells from the E18.5 mouse brain generated with the 10X Chromium [9] provides an opportunity to explore the relationship between population structure and the number of sampled cells necessary to reveal the underlying diversity of cell types. We validate the findings from this initial dataset in a recently published dataset of a variety of mouse organs [5]. Here, we present a framework for how researchers can evaluate whether a dataset has reached saturation, and we estimate how many cells would be required to generate an atlas of the sample analyzed here. This framework can be applied to any organ or cell type-specific atlas for any organism.

## Results

**Fig. 1:**
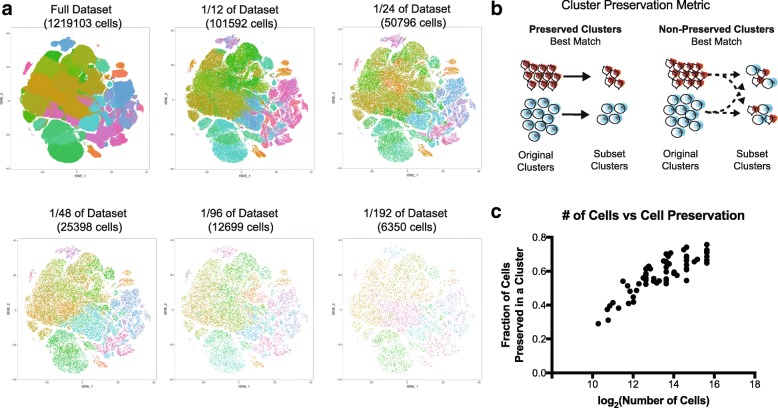
Downsampling of cell number preserves major cell type distinctions. **a** t-SNE plots of the full dataset and five smaller downsampled subsets. Each dataset is shown in the t-SNE space of the full dataset. Clustering was performed independently in every subset. **b** Cluster preservation is a key metric to evaluate similarities and differences between clusters from different analyses, measuring preservation as a fraction of the original cluster that remains in analyzed subsets. The diagram depicts a simplified cluster preservation calculation (see also the “Methods” section). **c** Cluster preservation represents the best instance of the fraction of a cluster that is represented during downsampling. Nine original subsets are represented and a total of 56 datapoints are represented; the cell number is shown on a log_2_ (number of cells) score to improve ease of graph interpretation

10X Genomics recently generated an open access dataset of 1.3 million cells captured and sequenced from an E18.5 mouse brain [10]. After performing quality control on the full dataset, we created randomized data subsets starting at 100,000 cells and subsampled by a factor of two down to a smallest data size of approximately 6000 cells (Additional file 1: Figure S1a). To account for potentially pervasive batch effects in single-cell datasets [11, 12], each subset was randomly selected from the libraries represented in the original dataset, and Louvain-Jaccard clustering generated clusters that each represented many of these libraries [7] (Additional file 1: Figure S1b–d). Visualization of each of these clusters in the space of the original dataset recapitulated the structure of the 1.3 million cells (Fig. 1a) and the proportional number of cells derived from each cluster verified that cells from all clusters were represented in every subset (Additional file 1: Figure S2a).

To compare clusters from the downsampled subsets to the clusters in the original dataset, we devised a cluster preservation metric. This metric examines how cells from the original clusters are distributed in the re-clustered subsets; the highest fraction of similarity defines the level of cluster preservation (Fig. 1b). In order to explore how much cluster preservation is achieved with subsets, we scored the cluster preservation for subsets containing variable numbers of cells and observed a plateau at around 0.70, emerging around 25,000 cells (Fig. 1c, Additional file 1: Figure S2b). Interestingly, this same number was the ceiling for cluster preservation regardless of reference subset, suggesting that 30% of cells are not systematically assigned to the same cluster. These in-between cells have been previously observed in other datasets [8]. We compared our cluster preservation approach to the Rand index [6, 13] and to the clusterRepro R package. The Rand index is a comprehensive number to indicate across all clusters how well they are preserved in two analyses and correlates well with cluster preservation when clusters are very similar, but across lower preservation scores, the cluster preservation metric described here stratifies results more effectively (Additional file 1: Figure S2c). The clusterRepro package did not correlate well with the cluster preservation score, likely a result of its design to compare data from different studies (Additional file 1: Figure S2d). These two established cluster preservation methods are optimized to datasets that are not single-cell based, and therefore may find less similarity between often noisy single-cell data clusters, but also demonstrates that the cluster preservation strategy presented here is skewed to find more similarities between clusters than these methods. These comparisons do indicate a role for a cluster preservation metric such as the one designed here that is intended for cell type-specific cluster comparison of single-cell RNA-sequencing data. Furthermore, the results of our cluster preservation analysis suggest that additional cells are not useful towards recapitulating original clusters, although these findings cannot inform the “accuracy” of either clustering solution.

**Fig. 2:**
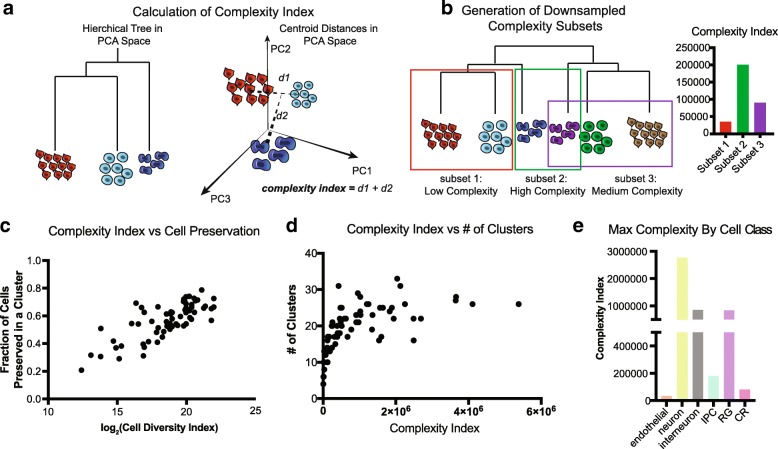
Downsampling of cell complexity preserves major cell type distinctions. **a** Cell complexity is calculated in the PCA space of the largest reference cell set analyzed. A hierarchical tree of clusters is calculated for each subset in the PCA space, and the total distance between the branches defines the cell complexity (see also the “Methods” section). **b** Cell complexity downsampling was performed by selecting branches of a larger tree with varied cell numbers and distances between groups. **c** Plot of complexity versus cell preservation. Each dot represents a point from 9 original subsets and a total of 56 datasets are analyzed. Log_2_ (cell diversity index) is used to easily interpret the dots at lower cell diversity numbers. **d** Number of clusters derived from subset analyses as a function of cell complexity. The graph begins to plateau at a cell complexity of ~ 100,000, suggesting there is a maximal number of clusters that can be derived from a sample even as cell number and complexity increases. **e** Complexity calculated by cell class annotations show neurons are the most complex of the cell types retrieved

Different tissues or organisms may be more or less homogenous in terms of population structure, and understanding how sampling requirements differ depending on the complexity of a tissue is essential to designing effective sampling strategies. Previous analysis has suggested that deeper sequencing depth is required for more similar cell types, and that estimating the diversity of cell types within a tissue can help with estimating the required sequencing depth [14]. We therefore developed a complexity index calculation that enables us to evaluate how many different cell types likely exist in a dataset by calculating Euclidean distance between cluster centroids in principal component space (Fig. 2a). Because the brain is thought to be an organ comprised of particularly diverse cell types, this dataset affords a unique opportunity to explore the impact of cell population complexity on clustering and classification. We selected groups from a hierarchical tree of clusters from one of the 101,592 cell sets, intentionally generating higher and lower complexity subsets of varied cell numbers (Fig. 2b, Additional file 1: Figure S2c). While this complexity index is positively correlated to cell number, we did generate examples of lower complexity datasets with more cells (Additional file 1: Figure S2d).

We utilized the complexity index as an alterative to cell number, examining cluster preservation as a function of complexity. Similar to cell number, we find that the cluster preservation score plateaus at approximately 0.7, and above the complexity score of approximately 100,000, cluster preservation score did not increase (Fig. 2c, Additional file 1: Figure S2e). Interestingly, when mapping the number of clusters that are generated from an initial clustering analysis, the number of clusters similarly plateaus at the same complexity index (Fig. 2d, e). These data suggest that beyond a complexity of 100,000, limited additional information about the sample can be gained through clustering analysis.

**Fig. 3:**
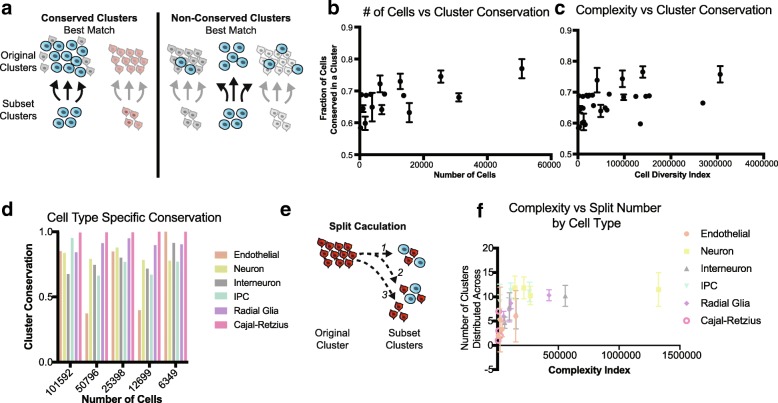
Cluster conservation from downsampled datasets. **a** Cluster conservation is an alternative metric to evaluate similarities and differences between clusters from different analyses, measuring conservation as a fraction of the subset cluster that originates from the same cluster. The diagram depicts a simplified cluster conservation calculation (see also Methods). **b** Cluster conservation as a function of cell number. Points are averaged within a sample from 56 downsampled subsets. **c** Cluster conservation as a function of complexity index. Points are averaged within a sample from 56 downsampled subsets. **d** When grouping clusters by cell type, cluster conservation is nearly perfect for most cell types. **e** The split of single cluster can be measured by counting the number of clusters that share ≥ 1 cell with either the original or subset cluster, as depicted in the diagram. **f** Cluster split number of subset clusters as a function of complexity index divided by cell type. Again, a plateau can be seen regardless of cell type around ~ 100,000. More complex cell types are split more, but complexity rather than cell type appears to indicate the number of splits that may occur

While cell preservation scores highlight how well original clusters are recapitulated, this assumes the original clusters are an accurate breakdown of the cells being analyzed. Alternatively, we devised a cell “cluster conservation” score that takes a bottom-up approach, examining how the subset clusters are represented in the original dataset (Fig. 3a). In general, cell cluster conservation scores are much more stable and increase only incrementally with cell number and cell complexity (Fig. 3b, c). Cluster conservation can be high either when a larger original cluster gets split into multiple clusters in a subset, or when original clusters are lumped into a single cluster in the subset, suggesting a broader utility for cluster conservation in identifying biologically meaningful cell classes (Additional file 1: Figure S3).

Broadly defined cell type designations are almost entirely conserved between downsampled sets and the original clustering solution (Fig. 3d). Exploration of the significant PCs in each of these subsampled sets indicated that for each of the 100,000 cell sets, PCs are highly correlated, suggesting that the major sources of variation are conserved even down to the smallest subset (Additional file 1: Figure S4). The high conservation of key sources of variation, including the gene networks defining cell types, likely explains why broad cell type assignments in the subsampled datasets remain largely the same even when substantially fewer cells are considered. However, the number of clusters in each of these initial analyses is smaller than the total number of clusters derived from the complete dataset. To identify additional subclusters, we performed iterative re-clustering (Additional file 1: Figure S5a–d), which substantially increased the number of clusters from each subset in proportion to the complexity of the parent cluster (Additional file 1: Figure S5e). Interestingly, iterative clustering highlights new sources of variation (Additional file 1: Figure S6), and the correlation of iterative PCA to the original PCA demonstrated a much smaller PC correlation.

Next, we compared clusters inferred from iterative analysis with the original clusters from the whole dataset. Surprisingly, we find that after downsampling, cells from individual clusters are reorganized into new clusters, but groups of clusters representing broad cell types are preserved (Additional file 1: Figure S3b). To quantify cluster stability, we measured the extent to which cells from every cluster were split by counting how many pairwise clusters contain cells from the cluster of interest (Fig. 3f). Intriguingly, this cluster split measurement plateaus at a complexity of about 100,000 regardless of cell type (Fig. 3g), suggesting that maximal recall of the whole dataset is capped at a cell number of ~ 25,000 and a complexity index of 100,000. These observations, together with the PC variance introduced by iterative clustering, strongly advocate for broad cell type classification followed by targeted enrichment and subtype characterization, especially in cases where the broad survey does not yield a large cell number of lower frequency cell classes.

In order to evaluate the applicability of these findings to other large datasets, we explored the next largest existing, published dataset of the mouse. This survey of multiple organ systems using a distinct technology called microwell sequencing generated profiles from 400,000 cells, of which 225,000 were high quality [5]. We similarly downsampled this data and explored the maintenance of cluster structure in tSNE space, cluster preservation, cluster complexity, and the cluster split. We similarly find that cluster preservation is high even at small cell numbers; this dataset is actually conserved even more completely with cell numbers as small as ~ 7000 cells, likely because the major cluster distinctions are driven by the organ of origin (Additional file 1: Figure S7). As such, we believe this validation analysis further supports the idea that a small number of cells can outline the structure of an atlas and more careful characterization by enrichment or depletion strategies as previously used [7] can more thoroughly complete the survey.

The metrics proposed here characterize the efficacy of varied downsampled subsets in recapitulating initial clusters, but none of the metrics indicate the sampling or clustering strategy most effective in recovering biologically interpretable clusters. To better understand the nature of downsampling, we focused our analysis on Cajal-Retzius (CR) cells, one of the lowest frequency cell types in the forebrain. CR cells are essential to the laminar organization of the brain [15, 16] and have been determined to originate from several sources within the brain that impart them with appropriate transcriptional markers of origin [17]. To explore this cell type, we isolated cells in the *Reln+*, *Tbr1+* cluster from the full 1.2 million cells dataset. By iteratively clustering these cells, we identified 18 distinct clusters with at least 10 marker genes distinguishing each cluster (Fig. 1a, Additional file 1: Figure S8a,b). The same process was applied to CR cells from each of the downsampled subsets originating from one 100,000 cells matrix.

Analysis of the clusters resulting from whole set iterative clustering suggested that some clusters were enriched for the highest and lowest levels of mitochondrial content as a fraction per cell which is frequently used as a quality control criteria [18] (Additional file 1: Figure S8c), and some had no unique identifiers separating them from other clusters, only a combination of marker level differences (Additional file 1: Figure S8d). Other clusters did have unique marker genes, though most genes were lost as markers through the downsampling process (Additional file 1: Figure S8e). However, two groups of clusters did highlight *Foxg1* and *Lhx9* [19, 20], markers indicating the putative developmental structure of origin. Violin plots of the expression of these genes in the full dataset and the downsampled sets show that while *Lhx9* maintains distinct cluster specific expression throughout downsampling, *Foxg1* loses cluster enrichment below 1/24th of the dataset (~ 25,000 cells, 815 CR cells). Additionally, exploration of an atlas of the developing mouse brain [21] shows that *Reln* is highly correlated to the genes that are preserved as cluster markers during some fraction of downsampling. (*Igf2*, *Satb2*, *Lef1*) (Additional file 1: Figure S8f) and there appears to be potential co-expression of these markers with markers of Cajal-Retzius cells based upon examination of the in situ hybrization (ISH) atlas images at E18.5 (Additional file 1: Figure S8g). Experimental work has already identified a subpopulation of dorsomedial *Igf2* positive Cajal-Retzius cells [22], and further experimental work will be necessary to characterize a functional role for these and the remaining uncharacterized subpopulations of Cajal-Retzius cells. However, the remaining, non-preserved cluster markers do not appear to show any potential overlap in these ISH images (Additional file 1: Figure S8g). Together, this may indicate that while a certain minimum number of cells is necessary to recover some cell type distinctions, not every cluster may be biologically relevant, although these data cannot prove a lack of existence of these clusters and additional validation may be required to firmly establish the number of Cajal-Retzius cell subtypes in the developing mouse. Instead, technical factors may influence variation during exhaustive iterative clustering, even after stringent quality control. Nonetheless, it is possible that the nine CR clusters from the full dataset without clear markers are biologically important. Similarly, CR clusters from subsets smaller than 1/24th of the dataset may have biological meaning, but we were unable to elucidate clear, meaningful distinctions.

**Fig. 4:**
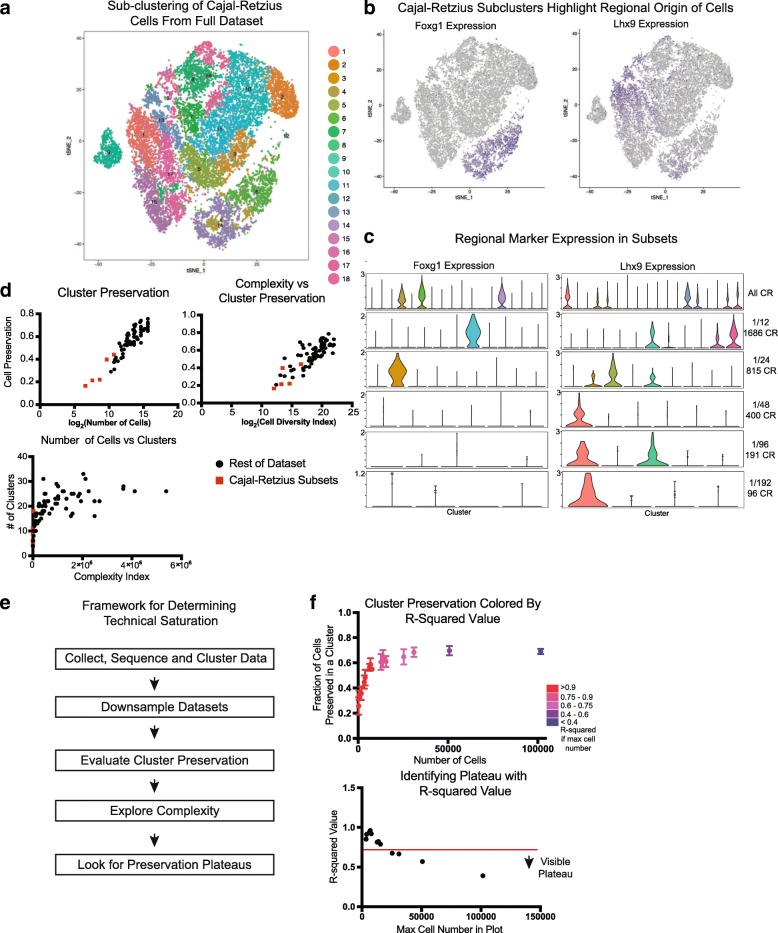
Downsampling of Cajal-Retzius cells. **a** t-SNE plot depicting the iterative clustering result of all 20,550 Cajal-Retzius (CR) cells from the full dataset. **b** Regional origin is a well-studied classifier of CR subtypes, and two of these markers feature prominently in the iteratively clustered dataset: *Foxg1* is enriched in three clusters while *Lhx9* is enriched in seven clusters. **c** Violin plots of regional markers in the full datasets and CR subsets of downsampled datasets indicate that these markers are enriched in one more clusters up until 1/24 of the dataset is sampled, after which *Foxg1* enrichment is diluted across multiple clusters. *Lhx9* enrichment is conserved to even the smallest downsampled subset. One subset for each downsampling is used. **d** Enrichment metrics of CR cells in the context of previously shown metrics indicate that informatically, saturation of this cell type has not yet been achieved. **e** Framework to evaluate if technical saturation has been achieved. **f** Examination of *R*^2^ values when incrementally decreasing the number of maximum cells used in the analysis shows that plateau emerges around an *R*^2^ value of 0.6

The CR subset offers an opportunity to explore how data subsets compare to the curves we developed with our various metrics. By plotting these CR subsets along the trajectories of cluster preservation, complexity, number of clusters, and split curves, we observe that by these metrics, the analysis has not yet reached saturation (Fig. 4d). However, our biological interpretation suggests that the main cell type subsets are identifiable within the subsets analyzed here. Our analysis provides a pragmatic analytical framework for evaluating whether a single cell dataset has been saturated. Specifically, downsampling of the dataset followed by implementation of the cell preservation score and complexity index analysis can reveal whether cluster diversity is saturated. If a linear regression on a plot of number of cells or complexity versus cluster preservation fits the downsampling with an *R*^2^ less than 0.6 and progressively decreases from larger cell number, saturation may be reached (Fig. 4e, f). This analysis suggests that saturation is much more quickly reached from the perspective of broad cell types, while iterative subtype identification is more fluid and requires careful biological validation.

## Discussion

Here, we present a framework for evaluating if cell atlas datasets have reached saturation by introducing a few simple and practical principles to evaluate whether enough cells have been sampled to characterize a heterogeneous tissue. Preservation, a metric for how well original clusters are preserved in data subsets, plateaus around 25,000 cells in the full dataset of 1.3 million cells from the E18 mouse forebrain. The plateau does not approach 1, but PCA beyond major subtypes likely introduces technical noise into the analysis, suggesting that clustering beyond this level of preservation may be an analytical artifact. This viewpoint is bolstered by the lack of biological insight and lack of distinct markers in clusters derived from iterative clustering of the Cajal-Retzius subsets. However, more experimental validation will be required to definitely validate the populations of cell types described here and in other atlas-scale studies.

We additionally present the complexity index, which scores the relative heterogeneity of a sampled cell population. We note that beyond a certain complexity, cell preservation also plateaus, at around 100,000 in our analysis. This plateau suggests that clustering has a maximal number of divisions that can be generated in a dataset per analysis, and supports a role for iterative clustering. However, even with iterative clustering, cluster preservation plateaus. This indicates that only a certain number of meaningful divisions can be identified within any dataset and that effective generation of the cell atlas will require multiple iterations of data collection, saturation analysis, and tissue level validation. In particular, efforts to either enrich for rare populations of interest or to deplete extremely common cell types will be required to effectively saturate all cell types that will be discovered in atlas-scale efforts. Using a low-coverage approach is likely an effective low cost, time-efficient strategy for surveying broad classes of cell types, and more high-coverage analysis will be required to more carefully characterize the subtypes or very low-frequency cell types within a given tissue. The analytical framework presented here provides a model for researchers to explore their own datasets and utilize data-driven strategies to evaluate when cell number and complexity have been achieved.

## Conclusions

Atlas-scale single-cell sequencing studies are becoming more common and well funded. These studies have the opportunity to offer insights into the cell composition of the human body and commonly used model systems such as rodents. Here, using cluster preservation and conservation metrics that we designed, an analysis of publicly available large-scale datasets indicates that for initial surveys, smaller numbers of cells will suffice and enable a larger cohort of individuals to be profiled before designing more in-depth studies that can characterize rare populations or particular cell types of interest. This study design strategy and the tools provided here may enable more efficient atlas-scale experiments and maximize the value of the cells profiled.

## Methods

### Data management

To access the full matrix of the 1.3 million cell set, we used the python instructions for accessing the HD5 object. The CellRanger software that is used to process 10X data outputs three files for genes, matrix, and barcode. Because of the size limitations of the full dataset, we broke the dataset into 64 parts and wrote these subsets to file. We then used the Seurat[3]Read10X command to assemble these three files in R. For each subset, we normalized the total counts per cell to 10,000 and eliminated cells with > 10% mitochondrial or ribosomal content as well as those with fewer than 1000 genes per cell. This quality control filtration reduced the total cell number in the analysis to 1,219,103. We evenly combined these filtered matrices into four parts, and then into two parts. Using a server node with 64 cores, a 2.6 GHz processor, and 512 GB of RAM, any additional sized matrices resulted in the error “problem too large”. Therefore, matrices of approximately ~ 100,000 cells were the unit primarily used in this analysis.

For the dataset from Han et al., we downloaded the .rds file from the Seurat tutorial page and selected the cells that met quality control thresholds of at least 1000 genes per cell. This resulted in a total of about 225,000 cells for downstream analysis.

### Downsampling

Random downsampling to the 101,592 cell sets occurred by repeated (> 10 times) shuffling of the two matrices of the full data, cutting them each in half, and recombining to make two random matrices. Once this had been performed, the 101,592 cell sets were generated in order from the full dataset, without replacement. Subsequent downsampling to smaller datasets resulted from random selection of this dataset; each subset formed the full set of available cells for the next downsampling (i.e., 50,796 cells were taken from one set of 101,592 cells, and the next subset of 25,398 cells was sampled from the set of 50,796 cells). Nine 101,592 cell sets were generated and analyzed for the sampling parameters included in the main figures.

For the Han et al. dataset, subsets began 112,949 cells, and variable genes were used for clustering analyses. In addition to repeatedly halving the dataset, 90%, 80%, 70%, 60%, 50%, 40%, 30%, 20%, and 10% of the data was used to generate datapoint for cluster preservation and complexity.

### Clustering

The full dataset was clustered by 10X Genomics using their CellRanger v1.2 graph-based clustering solution, loupe browser, and industrial scale computational resources. Clustering was performed on 100,000 cell sets and smaller using the graph-based Louvain-Jaccard method that has been previously described [7]. Briefly, fast PCA is performed on the full centered and scaled expression matrix for 50 principal components. Using the formula laid out by Shekhar et al., we calculated the number of significant PCs to be 18 and used this number of PCs for all comparative analyses. A nearest neighbors calculation is performed in the PCA space for 10 nearest neighbors, and from this analysis, a Jaccard distance was calculated for each pair of neighbors. This data is used as the input to the Louvain clustering. t-SNE plots were either generated in the PCA space of each analysis, or the loupe t-SNE or other clustered plots were used as the reference coordinates for a smaller dataset.

Iterative clustering was performed in the same way, but clusters of the same type were grouped together and used as the input to the clustering process. Hierarchical clustering was used to group clusters into a tree for distance calculations as input to the complexity calculation.

### Cluster preservation

Because this analysis uses subsets of cells with the same names as the analyses the clusters are begin compared to, cluster preservation was calculated by counting the number of cells that were the same in each pairwise cluster comparison. The fraction was calculated using the number of cells in the smaller dataset’s cluster. Preservation for each original cluster, then, was the maximum of the compared fractions across all subset clusters.

Example Perl code was used to generate a matrix of clusters vs clusters with the fraction of each cluster presented followed by an underscore and the fraction of the alternate cluster retained. The difference between the fractions is simply which analyses denominator is used for the calculation. The order depends upon the order of input files. Input is cluster identity file 1, cluster identity file 2, and output file name.

Code:
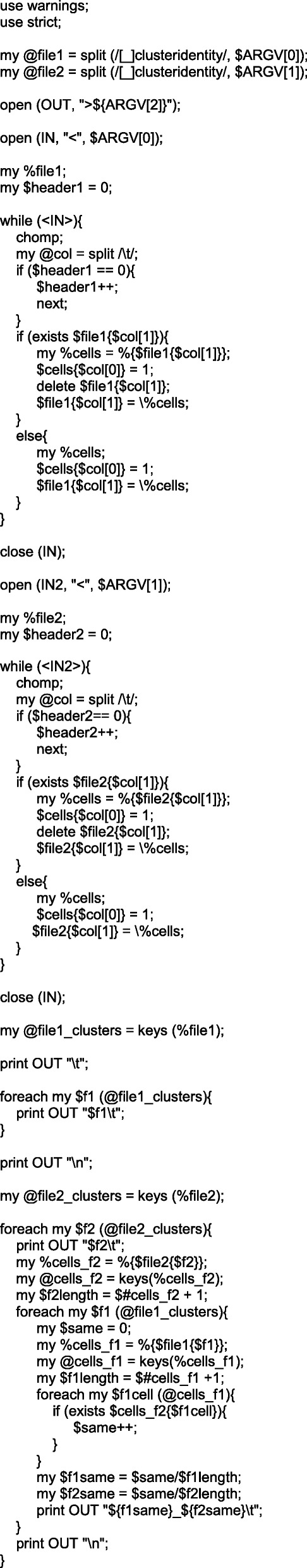


### Complexity index calculation

Complexity index is a way of measuring relative sample complexity by using Euclidean distances in the PCA space. For each set of clusters, the Seurat BuildTree function was used to hierarchically represent clusters in the PCA space of the cells being analyzed. Complexity index was calculated in the same PC space, so for a matrix of 100,000 cells, all downsampled sets were compared in a uniform PC space. To generate the index, the branch lengths of the tree were added based upon the centroid distances in the PCA space. The index is a number in arbitrary units. Complexity index scales positively with cell number, but it is possible to generate larger cell numbers with smaller complexity scores. Downsampling of complexity was performed by picking high and low complexity subsets from a tree of clusters from one of the 101,592 cell sets.

### Cluster conservation

Cluster conservation is a “bottom-up” evaluation of cluster preservation. Instead of observing how intact original clusters are in the subsets, cluster conservation measures the maximal correspondence in terms of the fraction of cells the same in pairwise cluster comparisons from the perspective of the new clusters. It uses the same calculation as cluster conservation but is the reciprocal analysis. Cluster conservation can be high even with low cluster preservation, particularly when there is a large imbalance of the number of clusters in the two analyses being compared.

### Split calculation

Measuring the number of splits is done by simply counting the number of pairwise clusters that have any cells from a cluster of interest, identifying how split up a cluster is. For example, even if cluster preservation is only 0.50, but its cluster split is 2, then it is a single cluster evenly split and could be considered a strongly preserved cluster. However, a cluster with preservation of 0.5 but a split of 10 would be considered to be much less preserved as its cells are found in a wide variety of subsequently generated clusters.

## Additional file

Additional file 1: **Figure S1.** Library and cluster composition metrics. **Figure S2.** Complexity index scales with downsampling. **Figure S3.** Schematic of conservation versus preservation metrics. **Figure S4.** Major sources of variation are preserved with downsampling. **Figure S5.** Major cluster features and subgroup determination. **Figure S6.** New sources of variation emerge in data subsets. **Figure S7.** Independent dataset validation of downsampling preservation. **Figure S8.** Cajal-Retzius cell diversity. tSNE plot of 20K CR cells from whole dataset colored by *Reln* and *Tbr1* expression indicating the clustering isolated canonically marked CR cells. (PDF 11394 kb)
