# Supplementary material for: Identification of cell types in a mouse brain single-cell atlas using low sampling coverage
Source: BMC Biol. 2018 Oct 11;16:113. doi: 10.1186/s12915-018-0580-x (PMC6180488; doi:10.1186/s12915-018-0580-x)
Supplement: Supplementary file 1 — Figure S1. Library and cluster composition metrics. Figure S2. Complexity index scales with downsampling. Figure S3. Schematic of conservation versus preservation metrics. Figure S4. Major sources of variation are preserved with downsampling. Figure S5. Major cluster features and subgroup determination. Figure S6. New sources of variation emerge in data subsets. Figure S7. Independent dataset validation of downsampling preservation. Figure S8. Cajal-Retzius cell diversity. tSNE plot of 20K CR cells from whole dataset colored by Reln and Tbr1 expression indicating the clustering isolated canonically marked CR cells. (PDF 11394 kb) [file 12915_2018_580_MOESM1_ESM.pdf]

Supp Fig 1. Library and cluster composition metrics

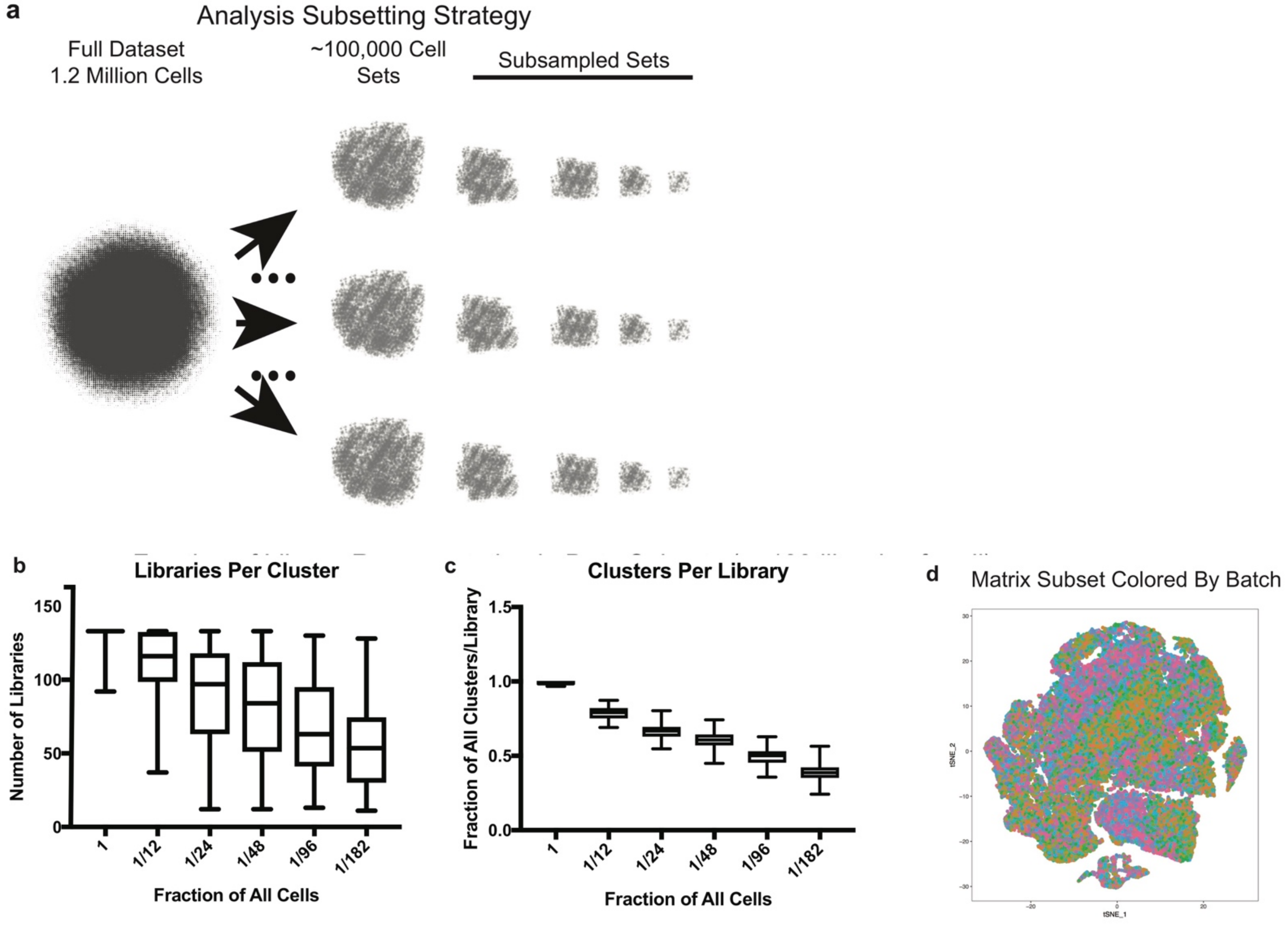

Additional File 1: Fig S1. Library and cluster composition metrics (**a**) Schematic depicting subsampling strategy for analysis (**b**) Number of libraries in a cluster across subsampled datasets. This number decreases as the dataset gets smaller, but the majority of clusters still have 10s of libraries even in the smallest set. (**c**) The fraction of clusters per library. All libraries contain a diversity of cell types, and much of this representation is preserved with downsampling. (**d**) tSNE plot of a matrix subset of 101,592 cells colored by the library of origin. Key of 133 libraries is not shown but each color is a distinct library and the libraries intermix thoroughly.

Supp Fig 2. Complexity Index Scales with Downsampling

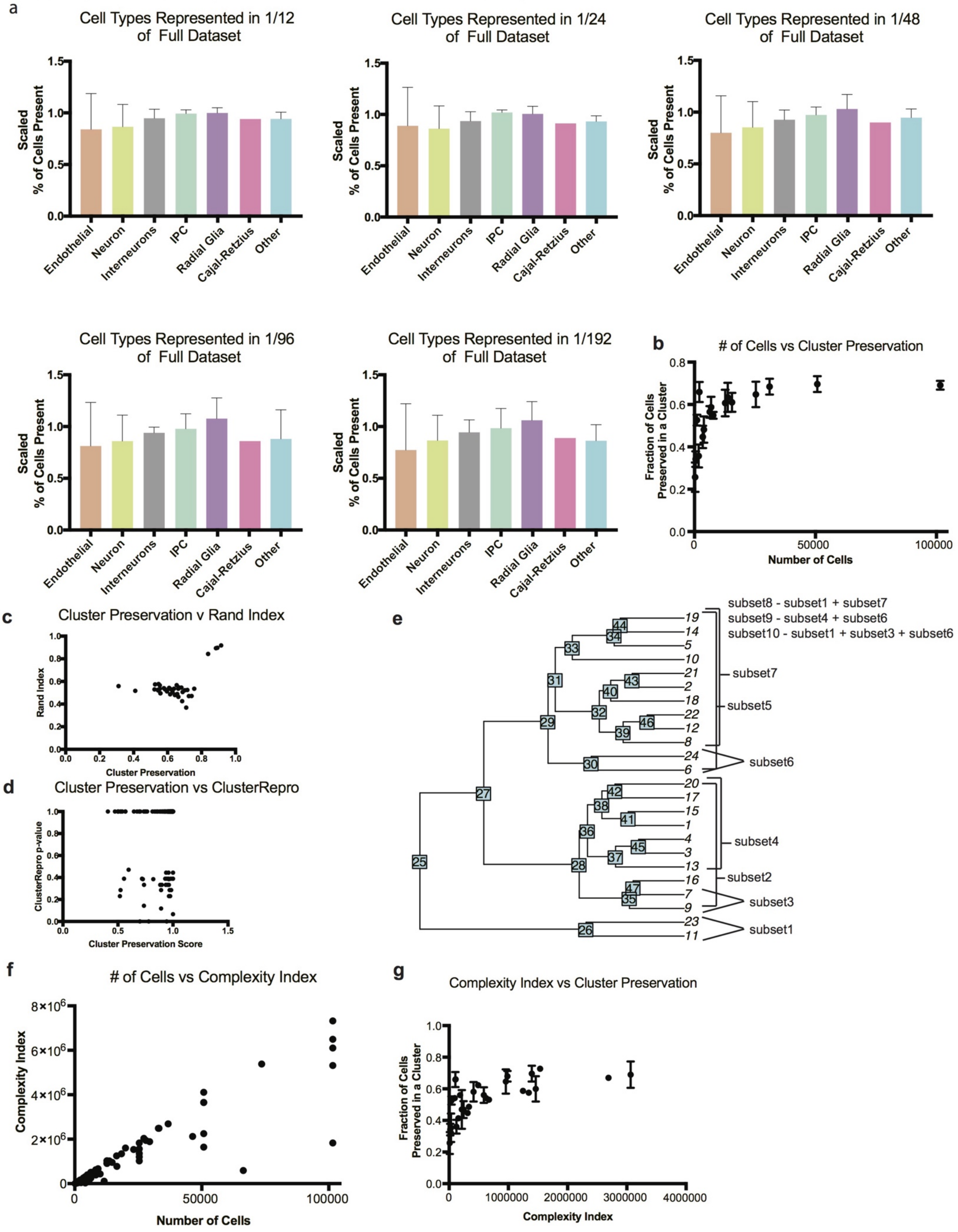

Additional File 1: Fig S2. Complexity index scales with downsampling. (**a**) Scaled proportion of cell types represented in each of the downsampled datasets. For the complete dataset, cell types were annotated by examining cluster markers and assigning cell type where possible. The number of cells from each of the full dataset represented in each subset was use to generate a fraction, and scaled to the proportion of the full dataset present in each subset. (**b**) Cluster preservation as a function of cell number. Points are averaged within a sample from 56 downsampled subsets. The graph begins to plateau at a cell number of ~25,000 cells. Similar cell number subset preservations were averaged and standard deviation error bars added; non-averaged graph is in Fig 1C. (**c**) Comparison of cluster preservation to published Rand index metric of cluster comparison. While they are correlated at high cluster preservation, the Rand index fails to capture loss of preservation. 9 subsets from the 1.3M downsampling and one subset from the MCA analysis were included in the analysis, with a total of 40 subsets analyzed. (**d**) Comparison of cluster preservation to clusteRepro package output based upon p-value result. The metrics are not highly correlated. (**e**) A hierarchical tree of clusters from one set of 101592 cells. This hierarchy was used to generate subsets of intentionally varied cell numbers and complexities. (**f**) Plot of number of cells versus complexity index. Cell number is correlated to complexity but complexity can be less in a larger number of cells, particularly when downsampling. (**g**) Cell preservation as a function of cell complexity. Points are averaged within a sample from 56 downsampled subsets. The graph begins to plateau at a cell complexity of ~100,000. For clarity, similar cell number subset preservations were averaged and standard deviation error bars added; non-averaged graph is in Fig 2C.

Supp Fig 3. Schematic of Conservation versus Preservation Metrics

a

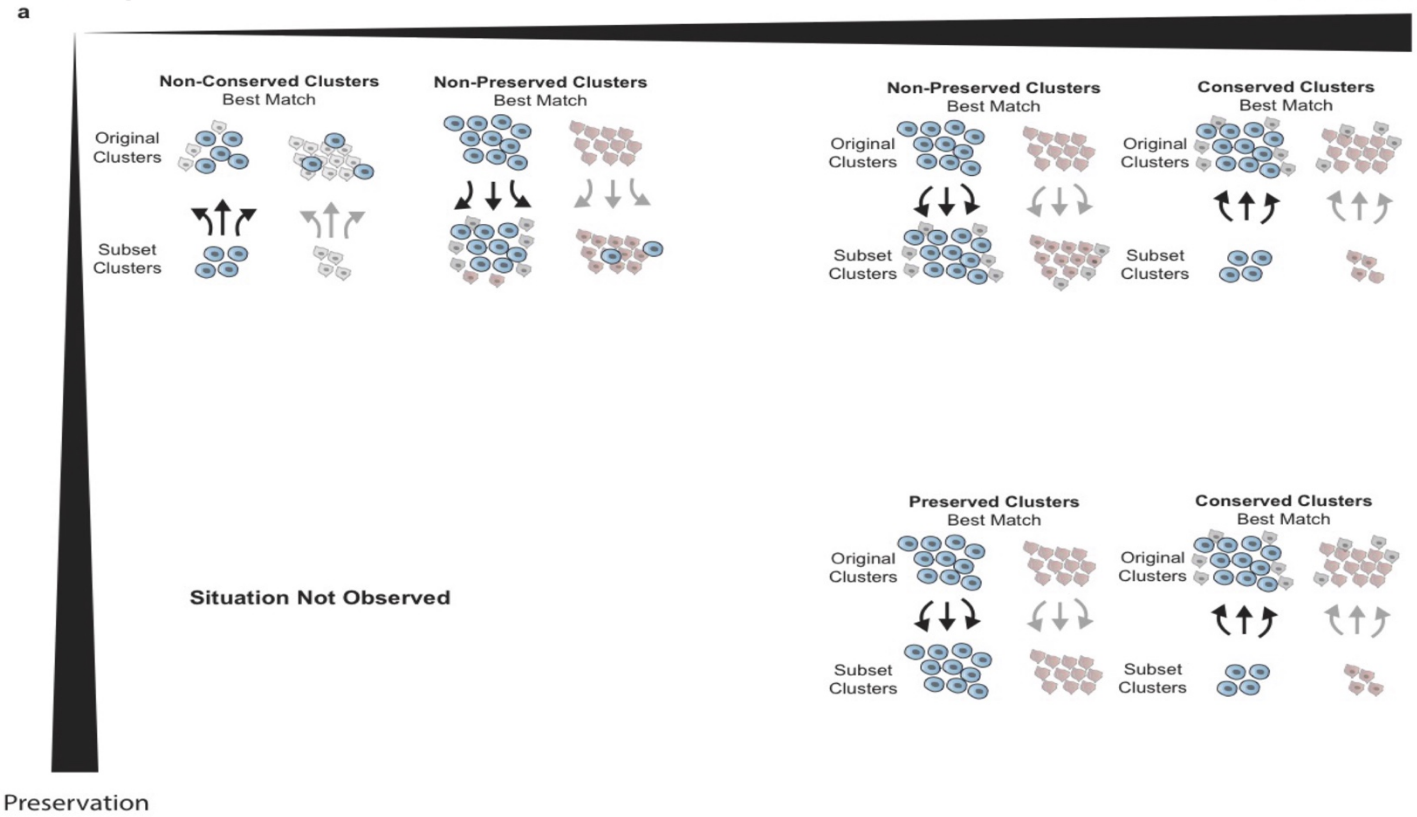

b

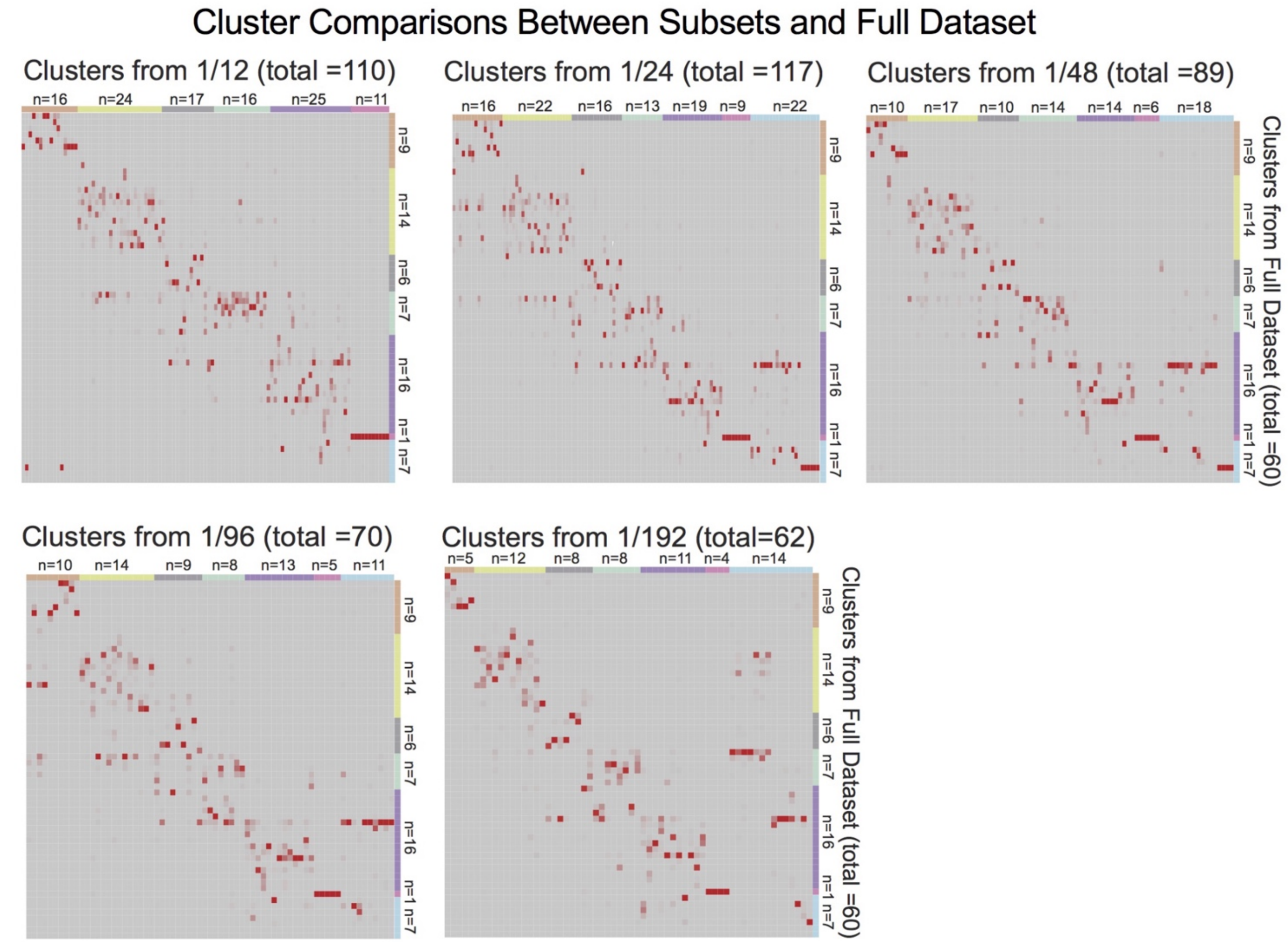

Additional File 1: Fig S3. Schematic of conservation versus preservation metrics. (**a**) Preservation and conservation are two concepts that can be used to measure cluster fidelity between datasets. Preservation is more dynamic and can range from low to high, but lower conservation is also possible. Schematic depicts the nature of the three intersections of preservation versus conservation that are observed in this dataset. (**b**) Heatmaps depicting the cluster conservation scores between each cell number subset and the full dataset. Blocks of conservation can be observed within each cell type, but iterative clustering results in a single cluster being split across multiple similar clusters. Interestingly, only one cluster of Cajal-Retzius cells was recovered from the full dataset, but analysis of each subset suggested that this population could be further divided into multiple clusters (see also Figure 4).

Supp Fig 4. Major Sources of Variation are Conserved with Downsampling

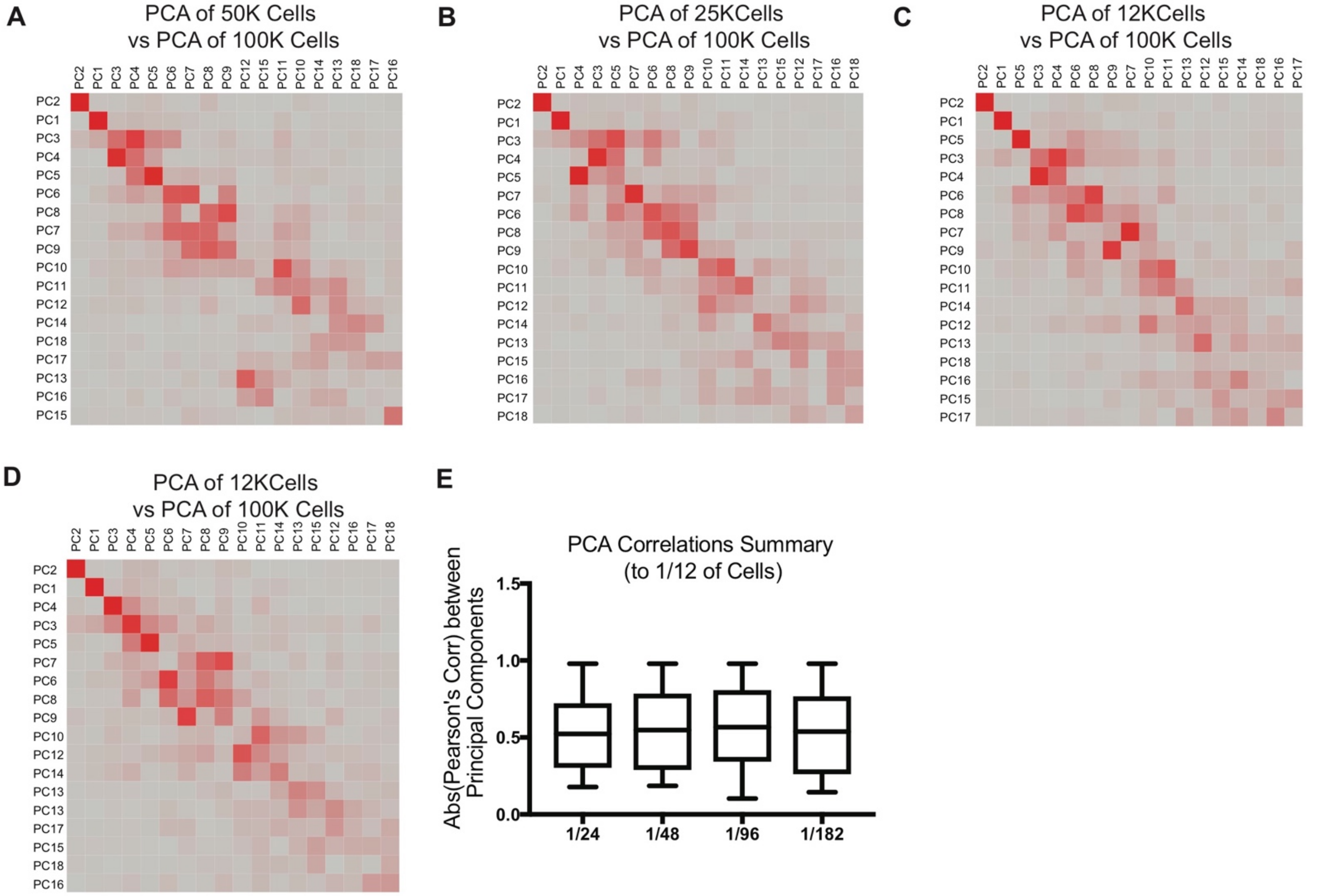

Additional File 1: Fig S4. Major sources of variation are preserved with downsampling. (**a-d**) When comparing the 100K cell matrix to further downsampled subsets, a strong diagonal across the matrix is observed. The PCA conservation, though not one-to-one, indicates strong principal component preservation across datsets. (**e**) Quantitative summary of the absolute values of the best PC correlations between the datasets explored here.

Supp Fig 5. Major Cluster Features and Subgroup Determination

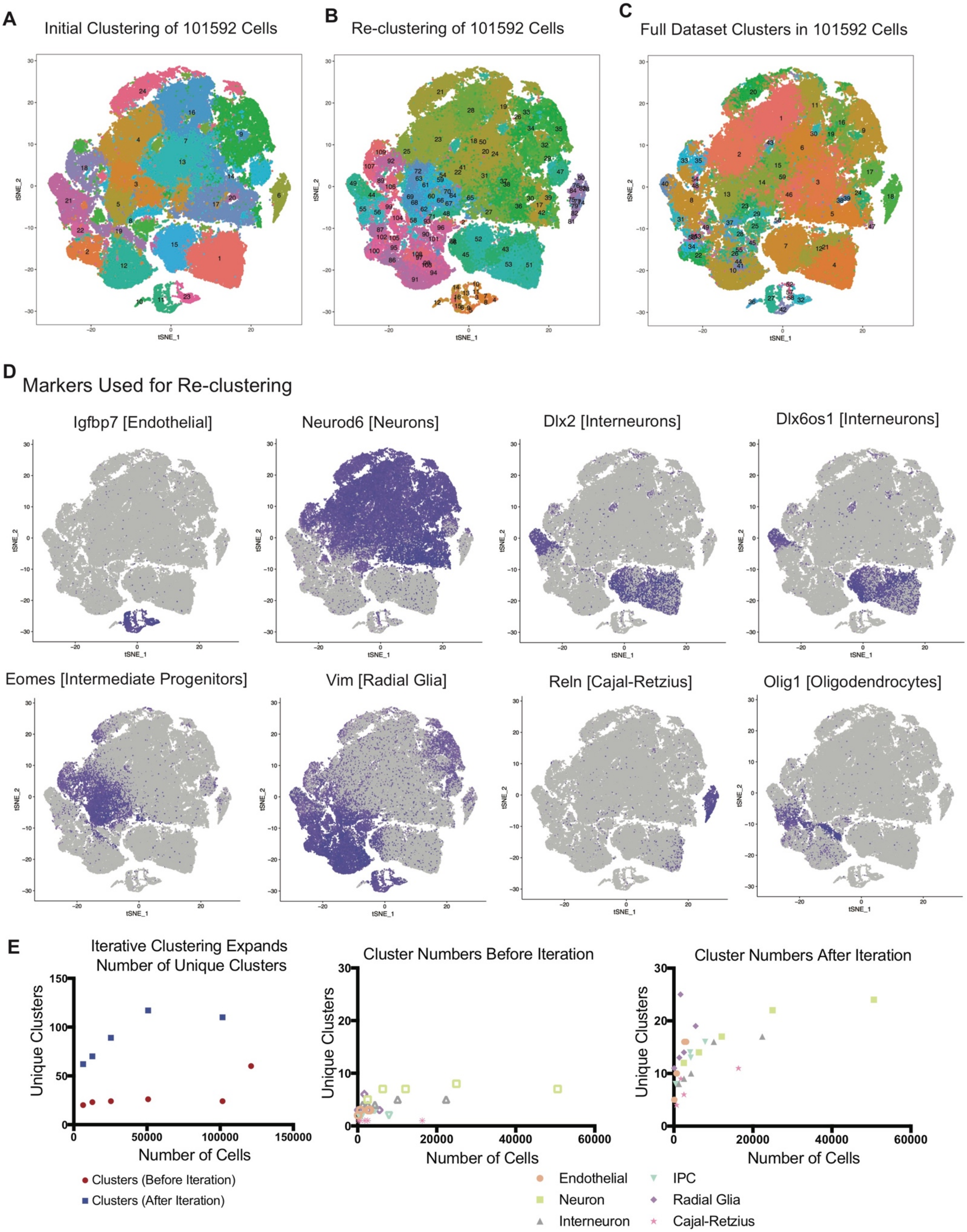

Additional File 1: Fig S5. Major cluster features and subgroup determination. (**a**) tSNE plot showing the initial clustering of one dataset of 101592 cells. (**b**) Using iterative reclustering, tSNE plot is colored by new clustering analysis. (**c**) Using the cluster designations of the cells used in this subset, clusters are colored on tSNE plot by their loupe cluster annotations. (**d**) Cluster markers used to designate clusters for iterative clustering analyses. (**e**) Plots of number of clusters identified before and after iterative clustering analyses. The number increases to a point, but the maximum is actually seen at 50K cells. Segregating this representation by cell type indicates that additional cluster resolution is dependent upon the number of cells in the subtype being re-clustered.

Supp Fig 6. New Sources of Variation Emerge in Data Subsets

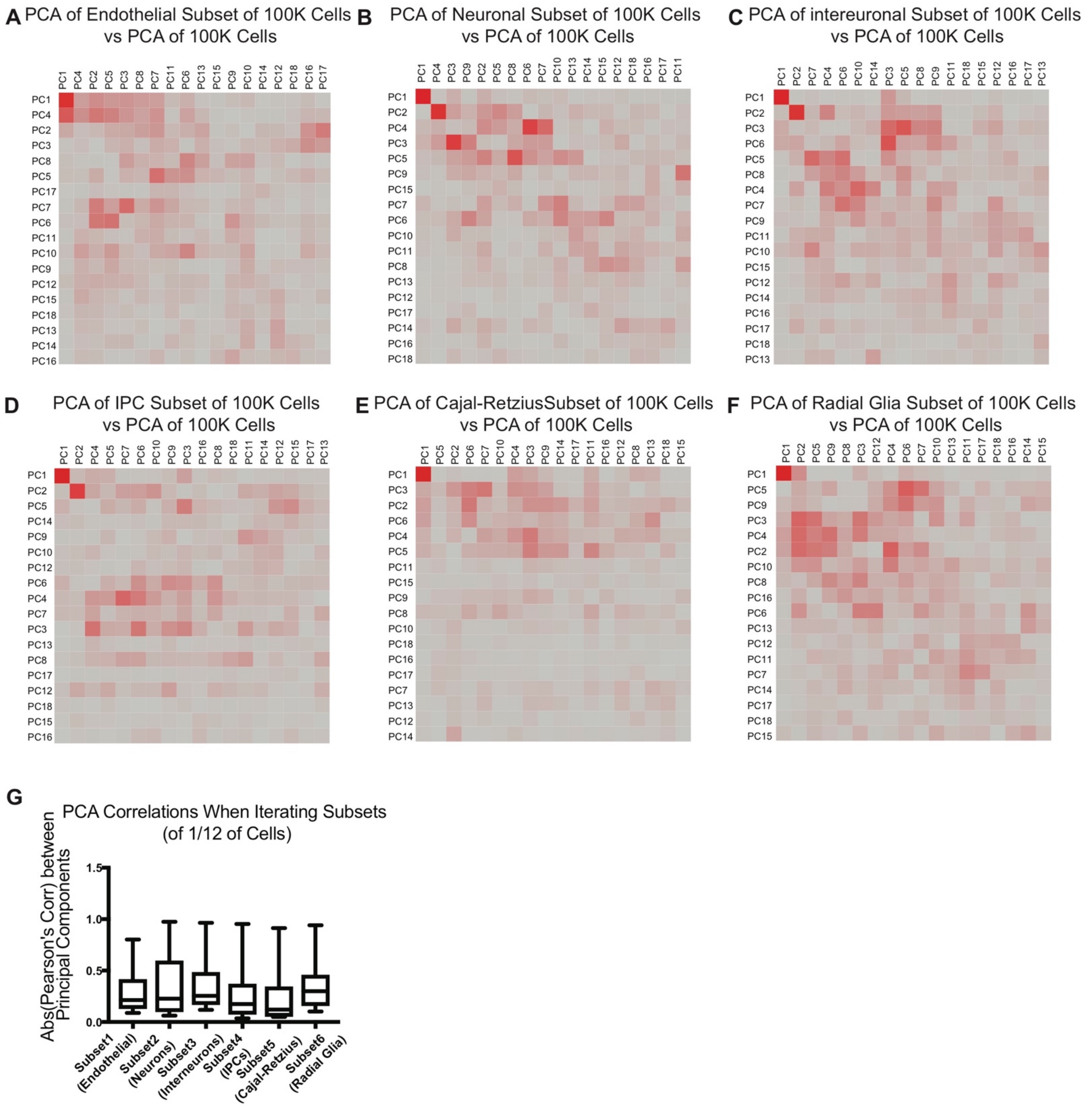

Additional File 1: Fig S6. New sources of variation emerge in data subsets. (**a-f**) Examination of PCA correlations between the whole dataset of 101592 and the cell type specific subsets being analyzed shows that only one or two PCs are typically highly correlated to one another, indicated the iterative clustering introduces additional sources of variation. (**g**) Quantitative summary of the of the absolute values of the best PC correlations between the datasets explored here.

Supp Fig 7. Independent Dataset Validation of Downsampling Preservation

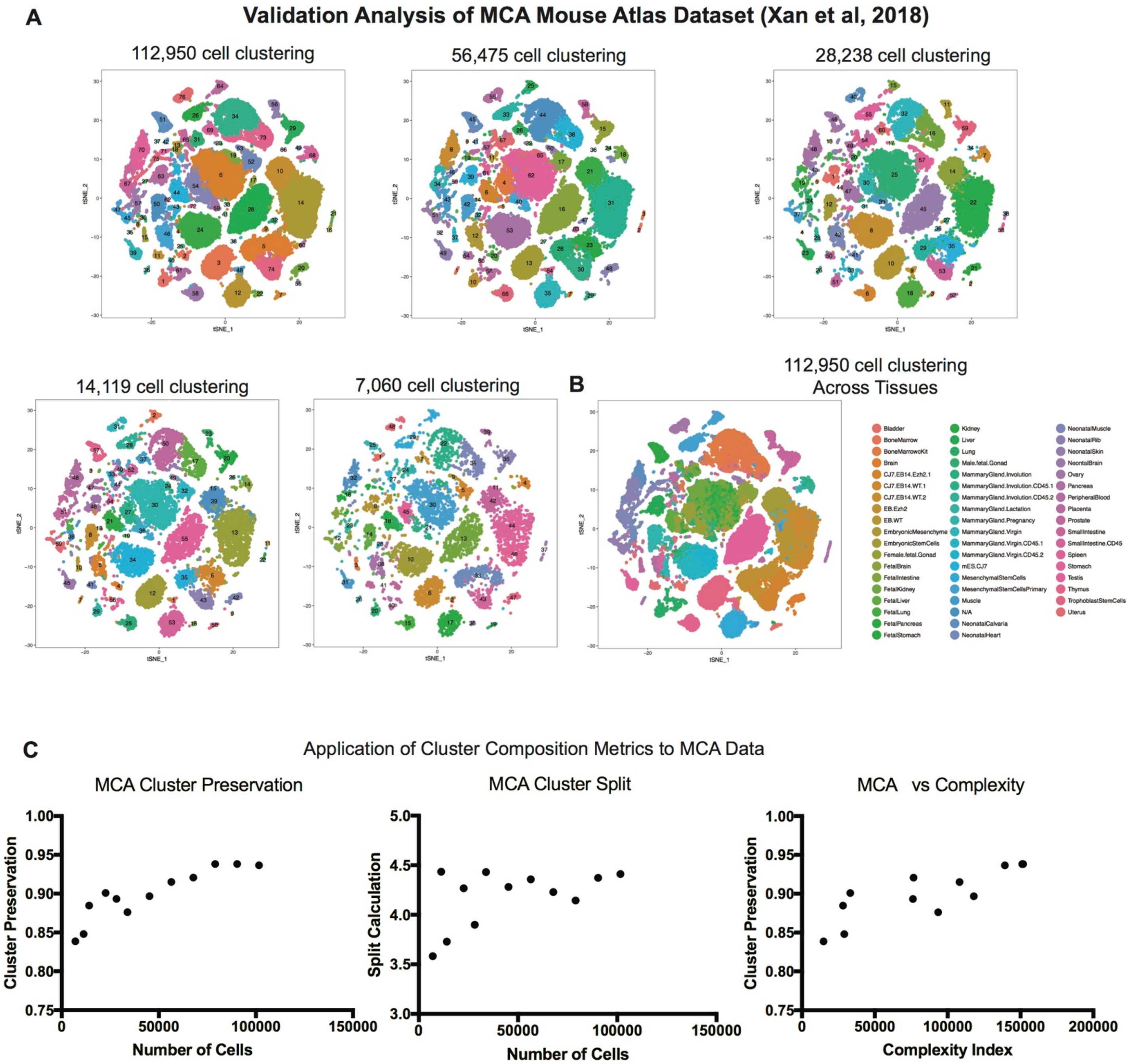

Additional File 1: Fig S7. Independent dataset validation of downsampling preservation. (**a**) Data from Han et al, 2018 was used for similar downsampling analysis as with the 1.3 million cell dataset. Exploring one dataset subset of 112950 cells and smaller downsampled sets shows very similar cluster structure in tSNE space. In each plot, the same tSNE coordinates are used but clusters are colored and labeled by the result from each individual clustering analysis. (**b**) The same tSNE plot depicted in the first panel of (a) is shown, but colored by organ of origin. As can be seen, most clustering is driven by organ of origin making this dataset distinct in composition from the 1.3 million cell dataset. (**c**) Examination of cluster preservation, cluster split, and complexity index shows a decrease of cluster similarity with downsampling, but in this dataset the overall preservation is much higher. This may be driven by the fact that most distinctions are organ based and can be observed with much smaller cell numbers (cluster preservation at ~7000 cells is still 83%).

Supp Fig 8. Cajal-Retzius Subcluster Comparisons to Downsampled Sets

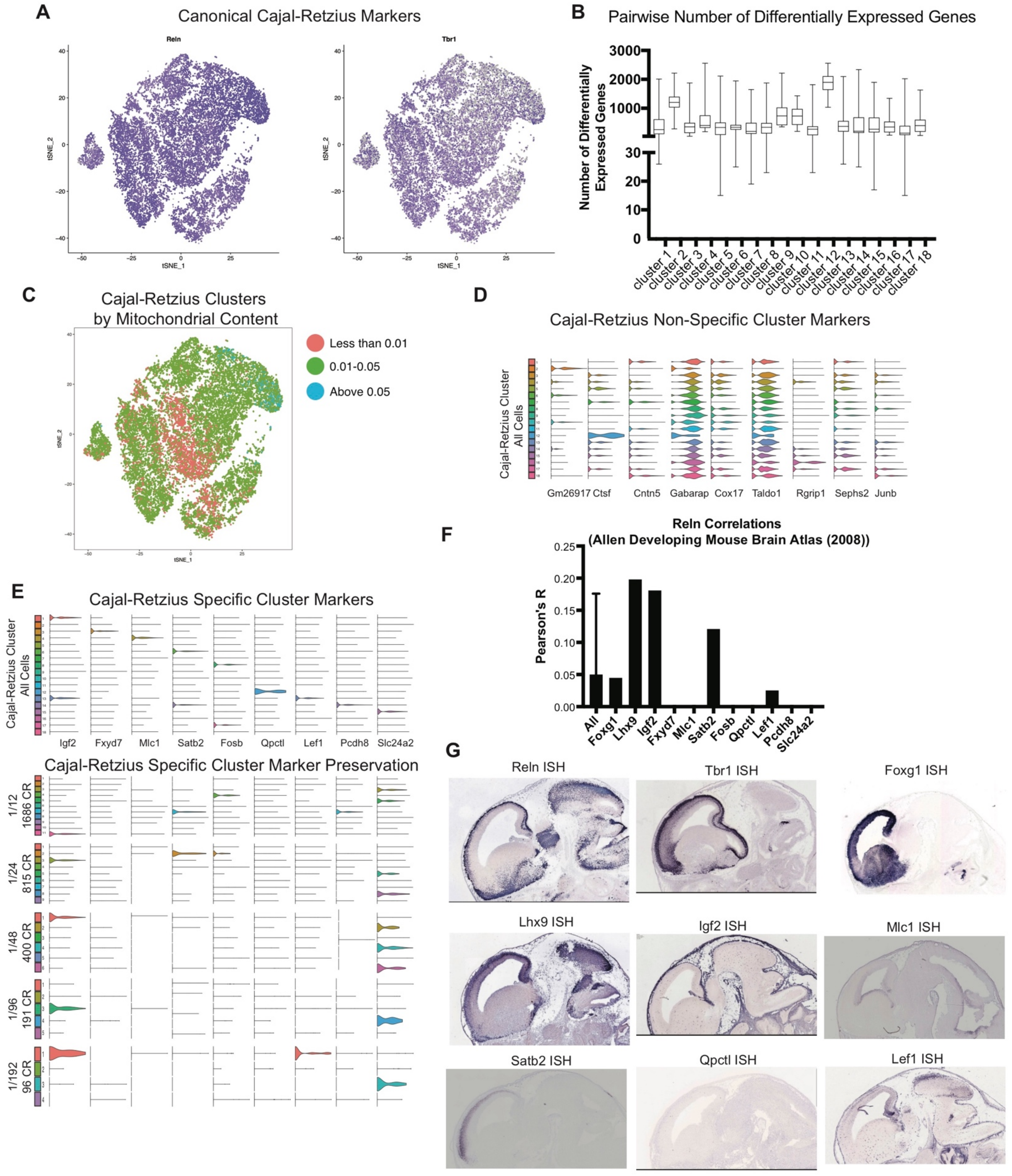

Additional File 1: Fig S8. Cajal-Retzius cell diversity. tSNE plot of 20K CR cells from whole dataset colored by *Reln* and *Tbr1* expression indicating the clustering isolated canonically marked CR cells. (**b**) Box plots of the number of differentially expressed genes between each cluster and all other clusters indicates that the 18 clusters identified are informaticaly distinct. (**c**) Coloring CR tSNE plot indicates pockets of enrichment of clusters dependent upon mitochondrial content, even after QC filtering. (**d**) Violin plots of non-specific CR cluster markers, these markers were the best enriched for some clusters but show expression across multiple clusters. (**e**) Specific CR cluster markers are shown in the top half, these markers are strongly enriched in one or clusters of the iteratively cluster 20K CR cells from the whole dataset. Examination of marker expression of these genes in downsampled CR sets shows *Igf2, Satb2, Lef1, and Slc24a2* are largely conserved with downsampling but other markers are lost, sometimes with the first downsampled set. (**f**) Histogram depicting the overall correlation values of correlated genes to Reln from quantification of *in situ* hybridization (ISH) of genes at E18.5 (Allen Developing Brain Atlas 2008). The known markers *Foxg1* and *Lhx9* are approximately at or above average correlation to *Reln*, while cluster markers such as *Igf2, Satb2,* and *Lef1* have similarly higher correlation to *Reln*. (**g**) ISH from Eurexpress (Diez-Roux et al 2011) similarly shows clear co-expression of known and novel markers *Foxg1*, *Lhx9, Satb2*, and *Lef1* to *Reln*, but other markers are not co-expressed at all. Sections are from E14.5 and attempt to include similar forebrain sections which visually corresponded to section 5 (*Foxg1, Tbr1, Lhx9)* and section 8 for the rest.
